# Dependence of Monocrystalline Sapphire Dicing on Crystal Orientation Using Picosecond Laser Bessel Beams

**DOI:** 10.3390/mi14040772

**Published:** 2023-03-30

**Authors:** Qiuling Wen, Jinhong Chen, Guoqin Huang, Changcai Cui, Dekui Mu

**Affiliations:** 1 Institute of Manufacturing Engineering, Huaqiao University, Xiamen 361021, China; 2 Xiamen Key Laboratory of Photoelectric Material Machining, Xiamen 361021, China

**Keywords:** sapphire dicing, picosecond laser Bessel beam, crystal orientation, mechanical cleavage

## Abstract

Dicing is a critical step in the manufacturing process for the application of sapphire. In this work, the dependence of sapphire dicing on crystal orientation using picosecond Bessel laser beam drilling combined with mechanical cleavage was studied. By using the above method, linear cleaving with on debris and zero tapers was realized for the A1, A2, C1, C2, and M1 orientations, except for the M2 orientation. The experimental results indicated that characteristics of Bessel beam-drilled microholes, fracture loads, and fracture sections of sapphire sheets were strongly dependent on crystal orientation. No cracks were generated around the micro holes when laser scanned along the A2 and M2 orientations, and the corresponding average fracture loads were large, 12.18 N and 13.57 N, respectively. While along the A1, C1, C2, and M1 orientations, laser-induced cracks extended along the laser scanning direction, resulting in a significant reduction in fracture load. Furthermore, the fracture surfaces were relatively uniform for A1, C1, and C2 orientations but uneven for A2 and M1 orientations, with a surface roughness of about 1120 nm. In addition, curvilinear dicing without debris or taper was achieved to demonstrate the feasibility of Bessel beams.

## 1. Introduction

Due to its excellent light transmittance, extreme hardness, and outstanding corrosion resistance, single-crystal sapphire is widely applied in semiconductor substrates, infrared military equipment, and optical windows [1,2,3,4,5,6]. With the development of manufacturing technology, the production cost of sapphire has greatly reduced, which makes it possible to apply it in more fields. Single-crystal sapphire has anisotropic material and has different crystal orientations. At present, A-, C-, and M-plane sapphires are widely used commercially. A-plane sapphire is mainly used in electronic products and superconductivity because of its advantages in hardness and permittivity. C-plane sapphire is mainly employed as an LED substrate and optical field due to its excellent light transmittance. M-plane sapphire has important applications in the growth of non-polar or semi-polar gallium nitride epitaxial films.

Cutting is an inevitable step in the manufacturing process for sapphire applications. For instance, a 2-inch LED wafer that contains nearly ten thousand LED devices needs to be separated apart to produce individual LED dies. The quality of the sidewall of cut sapphire is critical for the stability and efficiency of LEDs [7]. Conventional methods such as blade dicing [8], mechanical scribing, and breaking [9] were applied to separate LED dies. To realize a higher yield die count, extremely thin saw blades need to be used, which can easily break and wear. The kerf width of blade dicing is in the range of 50–250 µm [8,10], which is limited by the thickness of the blade. Suffering from the production of cracks, chipping, and delamination, mechanical dicing is also underwhelming.

Laser-based cutting technologies have the advantages of high efficiency, no tool wear, and being environment friendly. They mainly include laser full ablation cutting [10] and laser scribing and cleaving [11]. The laser beam is scanned along the contour line where the sapphire material is required to be separated. Laser ablation is performed layer by layer, deepening until the entire sapphire material is cut through. Laser full ablation cutting has the advantages of a simple process and low cost. However, large heat-affected zones cannot be avoided, resulting in chipping and cracks, which can lead to a reduction in the service life of a device [12]. The kerf width of sapphire generated by laser full ablation cutting is in the range of 30 to 80 µm [13]. The laser scribe-and-break method is performed in two steps: firstly, a laser scribes a groove in a line on the surface of sapphire sheets; subsequently, an external mechanical force is applied on the laser-scribed line to separate the sapphire sheets into two sections. In general, the kerf generated on the top surface of sapphire is 30–50 µm in width. Compared with laser full ablation cutting, the laser scribe-and-cleave method does not need to cut through the material, and the surface roughness of fracture sections of sapphire sheets can be less than 10 nm [14]. Nevertheless, numerous sputtering debris and cracks are still inevitable during the laser scribing process. In addition, sometimes material is not split along the laser-scribed line, and laser-cut sidewalls are inclined [15]. Whether it is laser full ablation cutting or the laser scribe-and-cleave method, multiple passes are required, which results in significant heat accumulation. Moreover, a Gaussian laser cannot achieve taper-free, high-aspect-ratio dicing due to its short focal depth. A Bessel beam is a non-diffracting beam with a long focal depth and small spot size, which is obtained by Gaussian beam shaping. Ultrafast laser has a small heat-affected zone and high machining accuracy, which makes it suitable for the precision machining of sapphire [16,17,18]. Thus, an ultrafast laser Bessel beam becomes an ideal light field for high-quality cutting of transparent and hard-brittle materials [19,20,21,22] due to the small kerf width, high efficiency, and high accuracy. Liao et al. [23] studied the effect of hole spacing, burst mode, laser power, and defocusing distance on the surface roughness of the cross-sections of silica glass cutting samples based on the picosecond laser Bessel beam. However, they did not investigate the effect of material anisotropy on cutting. Liu et al. [24] reported that crack formation inside single-crystal sapphire can be controlled by adjusting the pulse duration, single pulse energy, and scan direction of ultrafast Bessel laser beams. They achieved curvilinear cleaving of sapphire without debris or taper through the proposed crack control method, but they only used C-plane sapphire and did not consider the effect of crystal orientation. Li et al. [25] investigated the effect of scanning speed and sheet thickness on the sapphire dicing by using the femtosecond Bessel laser beam. And they pointed out that the key factors to achieving low sidewall surface roughness are the uniformity of Bessel beams and appropriate pulse separation. However, in their work, the crystal orientation of sapphire sheets is C–M 0.2°, and they also did not deeply study the influence of crystal orientation on sapphire dicing. However, the study on sapphire dicing along different crystal orientations using ultrafast Bessel beams and mechanical cleavage was not explored. Due to the different material properties of sapphire along different crystal orientations [26,27], it is necessary to study the influence of crystal orientation on sapphire dicing.

Here, we studied the dependence of sapphire dicing on crystal orientation using a picosecond Bessel laser beam drill and subsequent mechanical cleavage. First, a row of through microholes was drilled on A-, C-, and M-plane sapphire sheets along different crystal orientations by a picosecond laser Bessel beam. Then, mechanical loads were applied to the aligned microholes to separate sapphire sheets. The surface morphologies of the microholes and their surrounding microcracks on both the top and bottom surfaces of sapphire sheets were characterized. The fracture loads of sapphire sheets along different crystal orientations were measured. The surface morphologies and surface roughness of fracture sections of sapphire sheets were evaluated. In addition, curvilinear cleaving of sapphire was carried out to demonstrate the flexibility of the picosecond laser Bessel beam.

## 2. Materials and Methods

**Figure 1:**
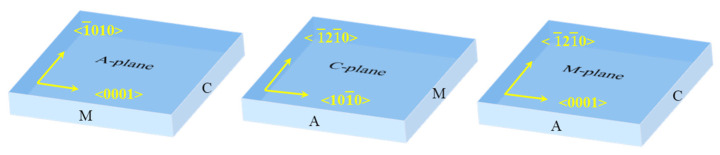
Schematic illustration of the crystal orientations of sapphire sheets.

The monocrystalline sapphire sheets were purchased from Wuxi Jingdian Semiconductor Material Co., Ltd., Wuxi, China. They were cut from 2-inch single-crystal sapphire wafers, which were double-sided polished to a surface roughness of less than 2 nm. The dimensions of the sapphire sheets are 10 mm long, 10 mm wide, and 430 μm thick. Figure 1 shows the schematic illustration of the crystal orientations of A-, C-, and M-plane sapphire sheets.

**Figure 2:**
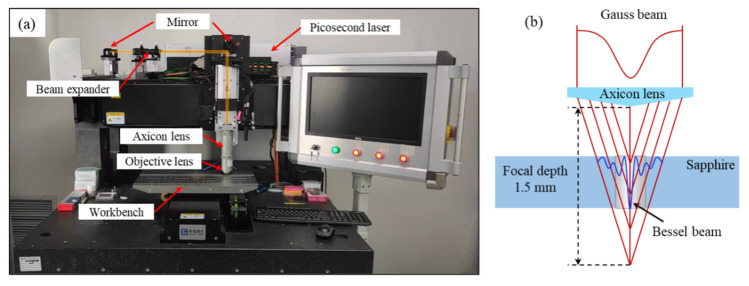
(**a**) Experimental setup, (**b**) Generation of Bessel beam.

Figure 2a presents the experimental setup, which mainly includes a laser device, optical system, and workbench. The laser device emits an ultrafast laser with a wavelength of 1064 nm, a pulse duration of 10 ps, a repetition rate of 40 kHz, and a maximum power of 60 W. The polarization of the laser beam was linear. The laser beam passed through two reflecting mirrors, a beam expander, and a fused silica axicon lens, and was then focused to form a spot with a diameter of about 2 μm via an objective lens. Figure 2b shows the formation of a Bessel beam. The Bessel beam was formed by Gaussian beam shaping through an axicon lens. The axicon lens has an aperture diameter of 25.4 mm, an apex angle of 176°, and a refractive index of 1.45. In the sapphire dicing experiment, in order to drill through microholes on the sapphire sheets, the laser pulse energy was set to 400 μJ, which was higher than the threshold energy determined by Liu’s method [28]. The laser scanning speed was fixed at 40 mm/s, and the scanning cycle number was 1. In addition, this work mainly aimed to study the effect of crystal orientation on the sapphire dicing by using Bessel laser beams. To make this work relatively concise, we did not study the influence of laser parameters on the microhole formation and subsequent mechanical breakage. However, ongoing work is being carried out to fill this gap.

In order to study the dependence of sapphire dicing on crystal orientation, a picosecond laser Bessel beam was scanned along different crystal orientations of A-, C-, and M-plane sapphires, as depicted in Figure 1. The laser scans along <0001> and <$10\bar{1}$0> crystal orientations for A-plane sapphire, along <$10\bar{1}$0> and <$\bar{1}$2$\bar{1}$0> crystal orientations for C-plane sapphire, and along <0001> and <$\bar{1}$2$\bar{1}$0> crystal orientations for M-plane sapphire. For simplicity, the laser scanning directions along <0001> and <10$\bar{1}$0> crystal orientations on the A-plane were called A1 and A2 orientations, respectively. Similarly, <$10\bar{1}$0> and <$\bar{1}$2$\bar{1}$0> crystal orientations on the C-plane were severally called C1 and C2 orientations, respectively; <0001> and <$\bar{1}$2$\bar{1}$0> crystal orientations on the M-plane were severally called M1 and M2 orientations, respectively.

The surface morphologies of Bessel beam-drilled microholes and Bessel beam-induced cracks around microholes on both top and bottom surfaces of sapphire sheets were observed using an Apreo S scanning electron microscope (SEM) made by Thermo Fisher Company (Waltham, MA, USA). Mechanical cleavage tests were carried out on an EZ-LX universal testing machine made by Shimadzu Co., Ltd. (Kyoto, Japan). The surface morphologies of fracture sections were examined by a Phenom II desktop SEM made by Phenom-Word Corporation (Eindhoven, The Netherlands). The three-dimensional (3D) topography and surface roughness of fracture sections were measured by the LSM700 Confocal Laser Scanning Microscope (CLSM) made by Zeiss Company (Oberkochen, Germany). The fractured sapphire sheets and curvilinear-cut sapphire samples were observed using a KH-8700 digital microscope made by HIROX Co., Ltd. (Tokyo, Japan).

## 3. Results and Discussion

### 3.1. Characteristics of Bessel Beam-Drilled Microholes along Different Crystal Orientations

**Figure 3:**
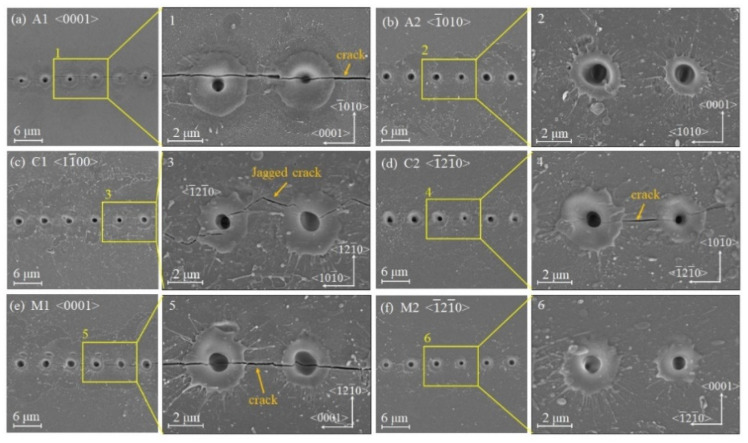
Top-view SEM images of Bessel beam-drilled microholes along different crystal orientations: (**a**) A1, (**b**) A2, (**c**) C1, (**d**) C2, (**e**) M1, (**f**) M2.

**Figure 4:**
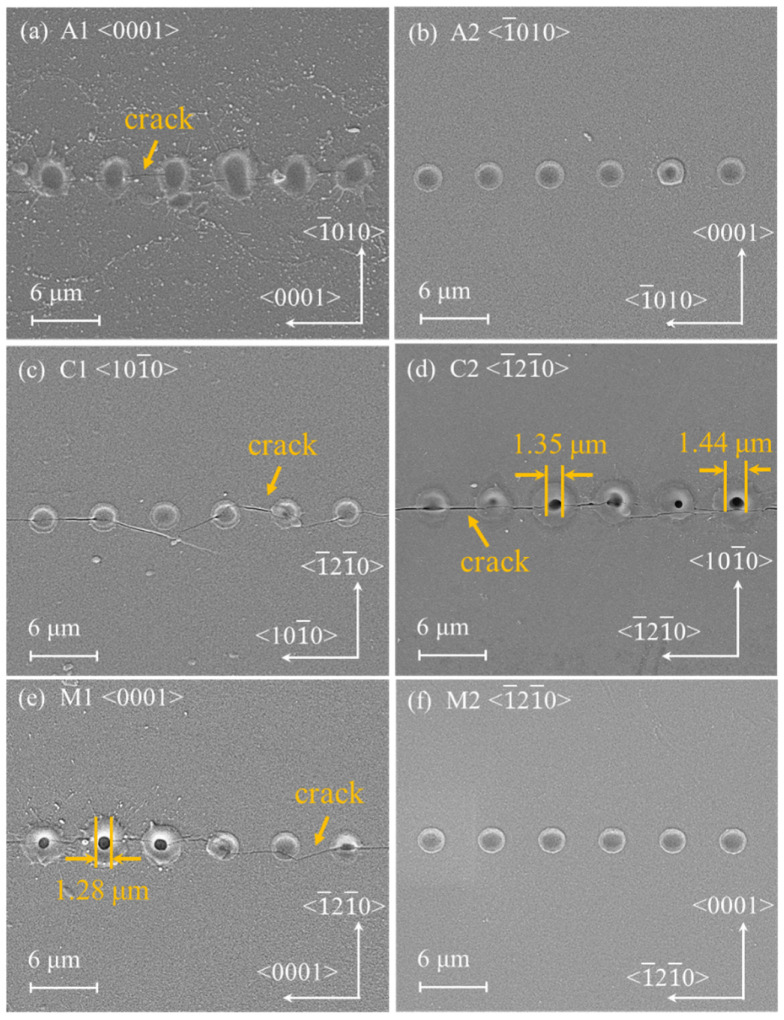
Bottom-view SEM images of Bessel beam-drilled microholes along different crystal orientations: (**a**) A1, (**b**) A2, (**c**) C1, (**d**) C2, (**e**) M1, (**f**) M2.

Aligned through microholes were drilled on A-, C-, and M-plane sapphire sheets along different crystal orientations by a picosecond laser Bessel beam, and their morphologies were characterized by SEM. Figure 3 presents the morphology of microholes on the top surface of the sapphire. As can be seen, all microholes are evenly arranged in a straight line on the sapphire surface, and their diameters are about 1–2 μm. The distance between two adjacent microholes is about 5 μm. Pronounced molten sputtering, and cracks appeared around the microholes. To see the details of cracks more clearly, Figure 3 (1–6) displays the magnified SEM images of the laser-drilled holes in the marked areas in Figure 3a–f. The characteristics of Bessel beam-induced cracks are closely related to crystal orientation. Along A1, C1, C2, and M1 orientations, cracks around microholes extended along the laser scanning direction, whereas no cracks were generated along the A2 and M2 crystal orientations. One can see that, for A1, C2, and M1 crystal orientations, cracks basically formed a straight line along the laser machining direction, while they were zigzagged along the C1 crystal orientation. In addition, cracks are wider and more pronounced for A1 and M1 crystal orientations. Figure 4 displays the morphology of microholes on the bottom surface of sapphire sheets, indicating Bessel beam-drilled microholes were penetrated from the top surface to the bottom surface. The diameters of the exit perforations are also 1–2 μm as marked in Figure 4. The cracks on the bottom surface were basically similar to those on the top surface. Compared with microholes on the top surface, microholes on the bottom surface had less sputtering. In addition, during the laser drilling process, the molten sapphire material was re-solidified inside and around the ablation areas, thereby blocking the outlet of some of the microholes.

### 3.2. Mechanical Cleavage of Sapphire Sheets along Different Crystal Orientations

**Figure 5:**
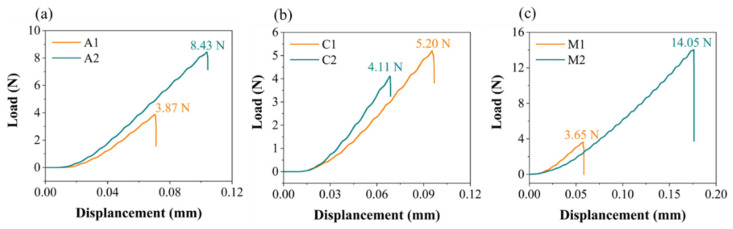
Variation curve of load with increasing displacement for A-plane (**a**), C-plane (**b**), and M-plane (**c**) sapphires along different crystal orientations.

**Figure 6:**
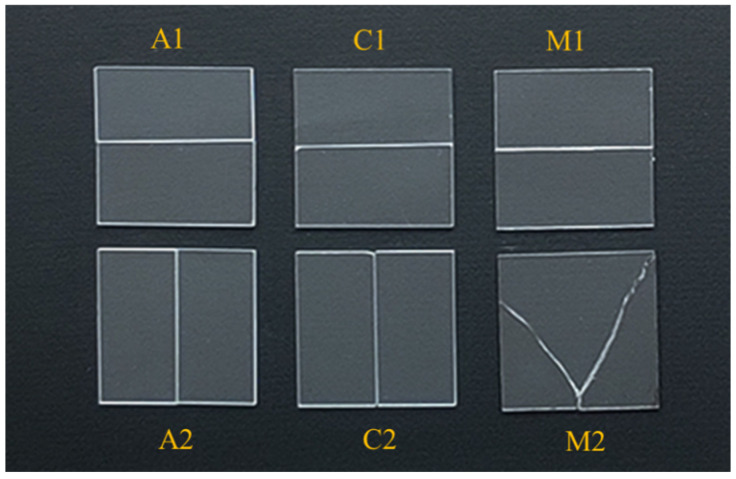
Optical image of fractured sapphire sheets along different crystal orientations.

In order to separate the sapphire sheets, mechanical loads were applied to the prefabricated microholes along different crystal orientations. Figure 5 shows the load-displacement curve for one test. As can be seen, the loading force increased with the displacement until a cliff-like drop occurred when the sapphire sheet was broken. To show a statistical study of the mechanical cleavage process, the sapphire cleaving test was performed three times, and the results are shown in Table 1. It is seen that the mean values of fracture loads along A1 and M1 crystal orientations are minimum, close to 3.9 N, followed by 4.64 N along C2 orientation and 6.32 N along C1 orientation, while the average values of fracture loads along A2 and M2 orientations reach a maximum value, about 12 N and 14 N, respectively. The difference in fracture loads along different crystal orientations is related to the laser-induced microcracks around the microholes. Cracks were generated around microholes for A1, C1, C2, and M1 orientations, except for A2 and M2 orientations. Along A1 and M1 crystal orientations, cracks spread in a straight line along the laser processing direction, and they were wider and more noticeable than those along other crystal orientations; therefore, the fracture loads were the smallest for sapphire breaking along A1 and M1 orientations. In addition, narrower cracks basically propagated in a straight line along the C2 crystal orientation, while the propagation direction of the crack was zigzag for the C1 crystal orientation. Therefore, it is more difficult to split sapphire along the C1 orientation than the C2 orientation. No cracks were generated for A2 and M2 orientations, so their fracture loads are greater than other crystal orientations. The reason is that the fracture sections along A2 and M2 orientations are both in the C-plane. According to the previous research [29], the fracture load of C-plane sapphire was significantly higher than that of other crystal orientations, which is consistent with our results. The optical image of fractured sapphire sheets is shown in Figure 6. It is clearly seen that, for A1, A2, C1, C2, and M1 crystal orientations, the sapphire sheets were fractured along the laser scanning direction. For M2 crystal orientation, the sapphire sheet was broken randomly and separated into three pieces. Therefore, dicing sapphire along A1, A2, M1, C1, and C2 crystal orientations using the proposed method could obtain satisfactory results, but sapphire sheets cannot separate along the M2 crystal orientation.

**Figure 7:**
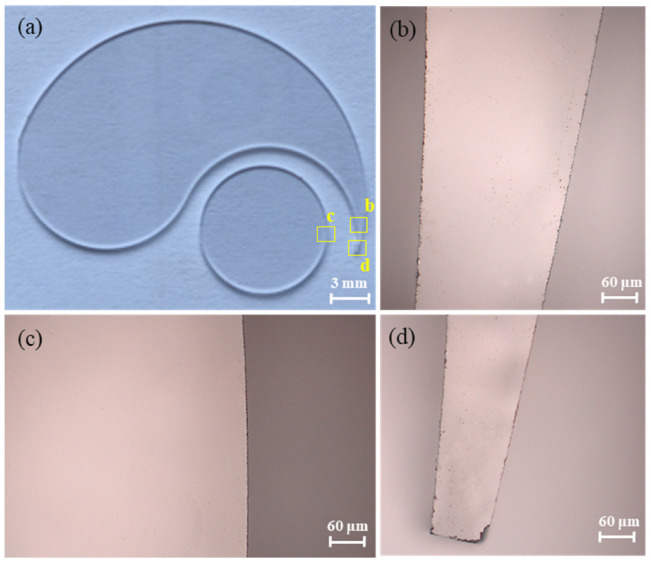
(**a**) Curvilinear dicing of sapphire, (**b**–**d**) Magnified views of cutting edges in (**a**).

In order to demonstrate the feasibility of this dicing method, curvilinear cleaving was carried out on C-plane sapphire sheets. Figure 7 shows the round and curved sapphire slices cut by the proposed approach. The radius of the circular sapphire sample was 5 mm, and the smallest distance between the two cutting tracks in Figure 7d was less than 100 μm. It can be seen that the cutting edges were uniform, with almost no chipping or cracking. In contrast to laser direct cutting, sapphire dicing using the proposed method has zero tapers and is crack- and chip-free. This reveals that precision cutting of sapphire can be realized using picosecond Bessel laser drilling rather than mechanical breakage.

### 3.3. Characterization of Fracture Sections along Different Crystal Orientations

**Figure 8:**
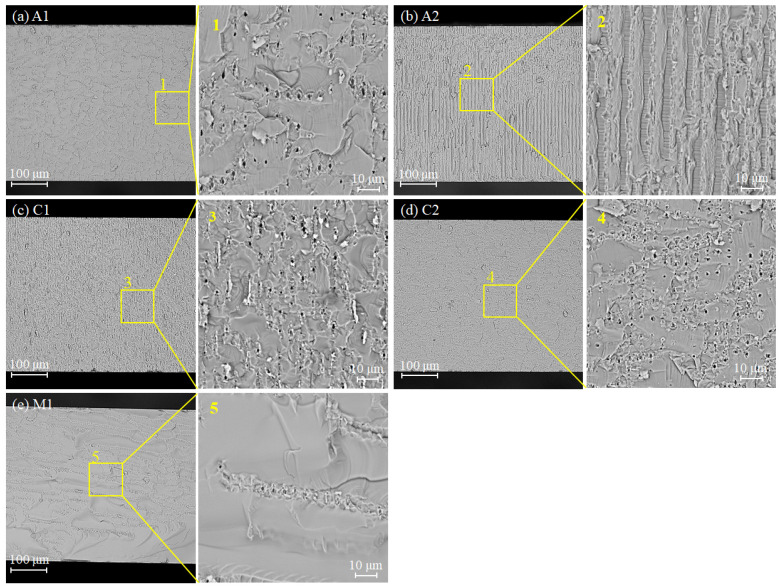
SEM images of fracture sections of sapphire sheets along different crystal orientations: (**a**) A1, (**b**) A2, (**c**) C1, (**d**) C2, (**e**) M1.

**Figure 9:**
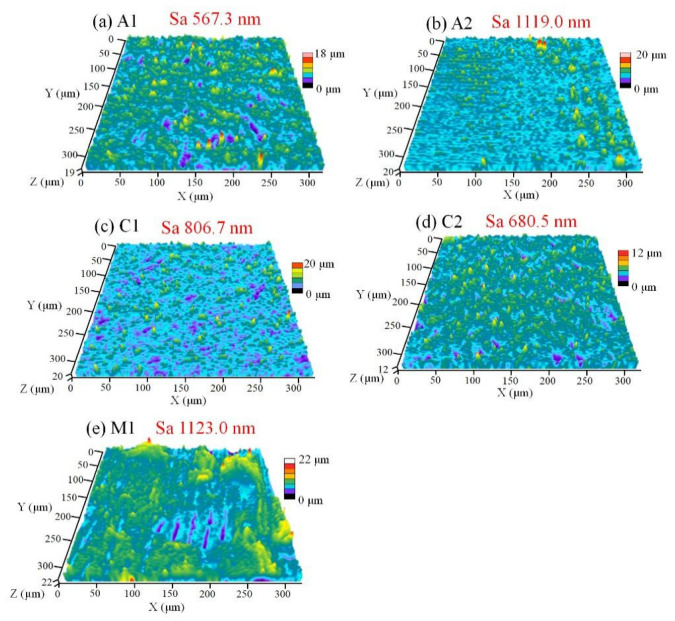
3D topography and surface roughness of fracture sections along different crystal orientations.

Figure 8 displays SEM images of fracture sections of sapphire sheets along different crystal orientations. The corresponding surface topography and surface roughness of the cut sections are shown in Figure 9. As shown in Figure 8a,c,d, the morphologies of fracture surfaces along A1, C1, and C2 orientations were relatively uniform. Dark pits that are randomly distributed on the fracture sections represent laser-modified areas. Other areas are separated by mechanical loading, which causes their surfaces to be similar. The surface roughness (Sa) of cross sections along the A1, C1, and C2 orientations was 567.3 nm, 806.7 nm, and 680.5 nm, respectively. For A2 orientation, periodic concave channels appeared on the cut section (see Figure 8b), which correspond to the laser-drilled microholes. The raised areas between two adjacent channels were obtained by mechanical tearing. The corresponding Sa is as high as 1119.0 nm. For the M1 direction, laser-modified areas on the cross-section of the sapphire cutting sample were connected in oblique lines (see Figure 8e). Between these modified layers, cleavage occurred randomly, and the Sa of the fracture section along the M1 orientation is 1123.0 nm (see Figure 9e). However, the dependence of the morphology of cut sections on crystal orientation is complicated and needs to be further systematically studied.

## 4. Conclusions

This study investigates the dependence of sapphire dicing on crystal orientation using picosecond Bessel laser drilling and subsequent mechanical cleavage. Through the proposed method herein, linear cleaving without debris or taper was realized for A1, A2, C1, C2, and M1 crystal orientations, except for the M2 crystal orientation. The experimental results show that the surface morphologies of Bessel beam-drilled micro holes, the fracture loads of sapphire sheets, and the surface morphologies of cut sections were closely related to crystal orientation. No cracks were generated around the microholes as the laser scanned along A2 and M2 crystal orientations, and the corresponding average fracture loads were 12.18 N and 13.57 N, respectively. While along A1, C1, C2, and M1 crystal orientations, Bessel beam-induced cracks extended along the laser scanning direction, and the corresponding mean values of fracture loads were obviously smaller than those along A2 and M2 crystal orientations. It is worth noting that, along M1 and A1 crystal orientations, the cracks are wider and propagate linearly along the laser scanning direction, resulting in a smaller fracture load of about 4 N. Therefore, the generation of cracks is conducive to mechanical cleavage. Furthermore, by characterizing the fracture sections of sapphire sheets, it was found that the fracture surfaces were relatively uniform when cleaving along A1, C1, and C2 orientations, whereas those along A2 and M1 orientations were uneven, as reflected by higher Sa values (1119.0 and 1123.0 nm, respectively). In addition, curved line cutting of sapphire sheets with no debris, chipping-free, and taper-free was realized to demonstrate the feasibility of ultrafast laser Bessel beams. This work may have instructive implications for the precision cutting of monocrystalline sapphire.

## Figures and Tables

**Table 1:** Average fracture loads of sapphire sheets along different crystal orientations.

Crystal Plane	Crystal Orientation	Fracture Load (N)			Average Fracture Load (N)
A-plane	A1	3.84	4.22	3.05	3.70
	A2	8.43	11.43	16.69	12.18
C-plane	C1	5.20	5.26	8.50	6.32
	C2	4.11	5.84	3.96	4.64
M-plane	M1	3.65	5.12	2.90	3.89
	M2	13.19	14.25	13.26	13.57

## Data Availability

All data related to the investigation process are stored personally by the authors; however, they can be shared with interested parties. All data can be formally requested from the corresponding author of the study.
